# Alone With the Kids: Tele-Medicine for Children With Special Healthcare Needs During COVID-19 Emergency

**DOI:** 10.3389/fpsyg.2020.02193

**Published:** 2020-09-09

**Authors:** Livio Provenzi, Serena Grumi, Renato Borgatti

**Affiliations:** ^1^Child Neurology and Psychiatry Unit, IRCCS Mondino Foundation, Pavia, Italy; ^2^Department of Brain and Behavioral Sciences, University of Pavia, Pavia, Italy

**Keywords:** COVID 19, telerehabilitation, children, developmental disabilities, family, parents, early intervention

The Coronavirus Disease of 2019 (COVID-19) is a contagious respiratory illness (Sohrabi et al., 2020) that following an initial outbreak in China is rapidly spreading worldwide. New positive cases are increasingly identified in a growing number of countries and the emergency has been recognized as a global pandemic (Coccia, 2020). To face and cope with such an unprecedented healthcare emergency, National governments have adopted specific strategies to limit the large-scale impact of the contagion (Parodi and Liu, 2020; Remuzzi and Remuzzi, 2020). Despite between-country differences exist (Chintalapudi et al., 2020; Roux et al., 2020; Tarrataca et al., 2020), these measures have generally changed from the initial attempts of containment to the subsequent mitigation actions. Lockdown acts have been largely adopted to slow the virus spread, to reduce the demands of intensive healthcare, and to control the contagion rate in the medium-long period (Parodi and Liu, 2020).

## The Fragile Condition of Families of Children with Disability During the Covid-19 Emergency

In this setting, the direct and indirect implications for citizens and healthcare specialists have been largely highlighted (Barello and Graffigna, 2020; Barello et al., 2020; Tian et al., 2020; Wang et al., 2020). Fragile and at-risk people—such as children with neurodevelopmental disabilities and their parents—are especially exposed to psychological stress related to the Covid-19 contagion and the lockdown (Provenzi and Tronick, 2020; Provenzi et al., 2020). A major consequence of the lockdown was the suspension of psychological and rehabilitation services for the healthcare and educational needs of children with neurodevelopmental disability (Schiariti, 2020; Thompson and Rasmussen, 2020). Although these children may present with different clinical conditions—e.g., autism spectrum disorders, psychomotor delay, genetic disorders and rare syndromes—they all share common special healthcare needs that require intensive interventions (Wilson et al., 2014; Järvikoski et al., 2015; Giusti et al., 2018).

Italy was hit first by the Covid-19 emergency among European countries (Remuzzi and Remuzzi, 2020) and—at the present moment—the count of positive cases is second only to the United Kingdom. Consistently, the Italian government faced the rapid and partly unexpected rise of the Covid-19 emergency with limited scientific, social and economic references. After the adoption of mitigation strategies characterized by general services lockdown, both public and local initiatives have recognized the needs of the most fragile individuals and specific supportive services have been developed (Boldrini et al., 2020; Leocani et al., 2020). Nonetheless, greater efforts are needed to meet the needs of the families of children with disability (Amaral and de Vries, 2020).

Following the lockdown, parents of children with the most severe neurodevelopmental conditions and with limited autonomy in daily activities may feel that they are left alone in caring for their kids (Dalton et al., 2020; Thompson and Rasmussen, 2020). The suspension of daily rehabilitation services and the lack of alternative recreational opportunities leave these parents alone in caring for their children, with the burden of balancing their psychosocial resources between caring for their child special needs and accomplishing their job duties. These families have to reinvent their own space and time organization, trying to find new ways to deal with their own needs and those of their children. Additionally, they may partially or completely lack the support of specialists—e.g., psychologists, educators, rehabilitation professionals, social workers—with which they are used to engage and from which they expect to receive solutions and relief.

## Family-Centered Tele-Medicine Interventions: Bridging Physical Distancing and Assuring Continuity of Care for Children and Parents

**Figure 1:**
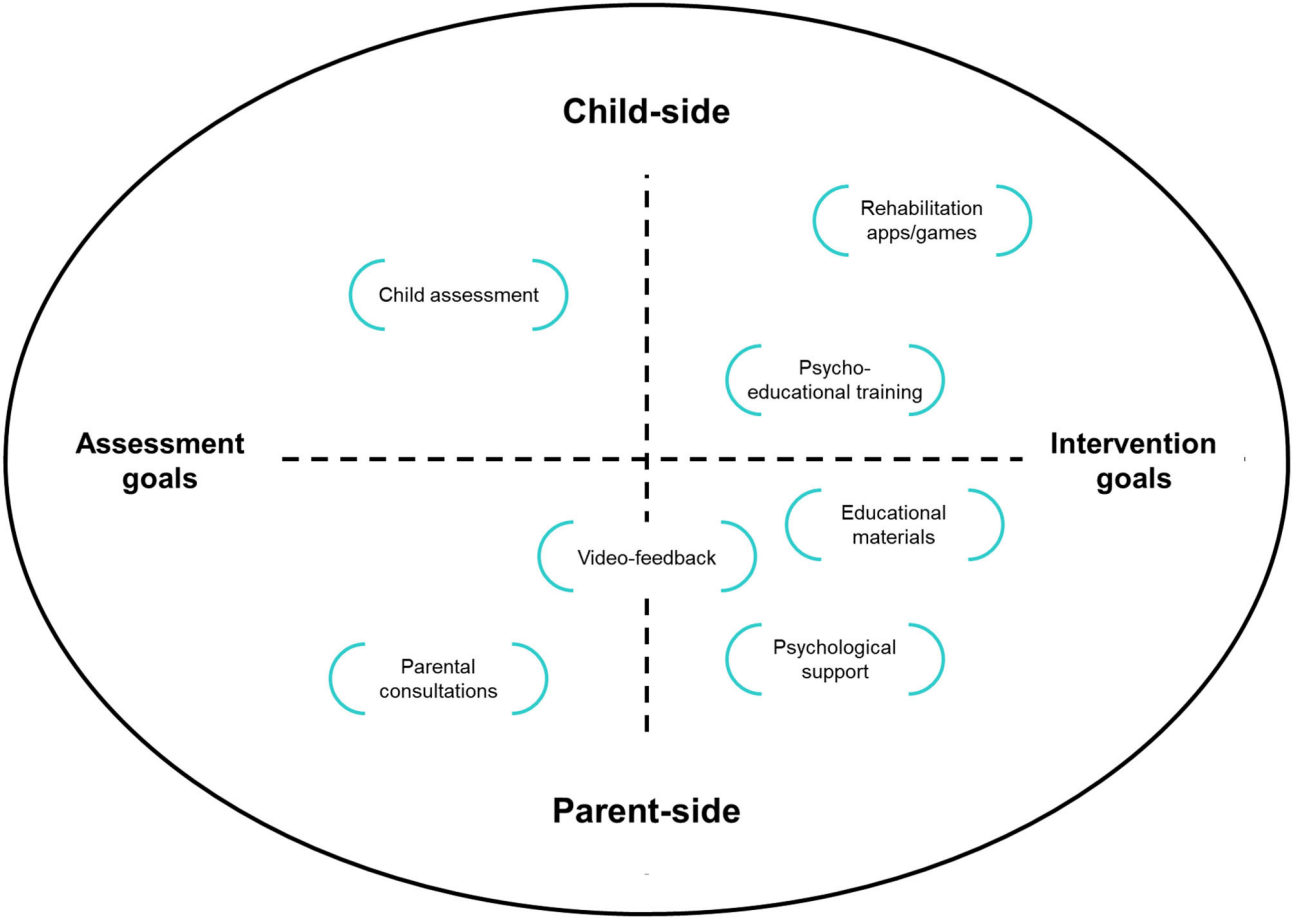
Examples of different telemedicine solutions defined in terms of focus (parents, children) and goal (assessment, intervention).

We live in a world where physical distances can be easily bridged by using telecommunication devices such as personal computers, smartphones, and tablets. In order to encounter and appropriately respond to the needs of parents of children with special healthcare needs, specialists should invest energy and resources in tele-medicine tools and strategies (Choon-Huat Koh and Hoenig, 2020). At the same time, policy makers and hospitals are requested to invest in interventions based on tele-medicine in order to manage the suspension of outpatient services and provide continuity of care (Veerapandiyan et al., 2020). The flexible nature of the technological support allow researchers and clinicians to develop tailored solutions that can serve different scopes: from assessment to intervention and from parent- to child-focused activities (Figure 1). The use of remote consultations, for example, both in the audio or video format, may be used for children behavioral assessment (Schopp et al., 2000; Barretto et al., 2006) and to communicate closeness, compassion and comprehension to parents while assuring quality of care, even if in the context of mobility constraints (Follmer et al., 2010; Vismara et al., 2013, 2018). Professionals can also share with the parents videotapes of intervention and rehabilitation sessions that they previously registered with the child, in an effort to provide visual examples and hints about how to pursue a continuation of care at home (Sourander et al., 2016). Specialists and parents can also use remote internet connections to share and co-create tools and materials to better explain the present COVID-19 situation to children with psychomotor delay or intellectual disability (Camden et al., 2019). Digital versions of augmentative alternative communication symbols may allow adequate access to COVID-19-related information to children with language impairments. Similar *ad-hoc* materials should be created and shared openly with the rehabilitation community (Langkamp et al., 2015). Telephone and video-chat connections can also serve the scope of providing parents and older children with psychological support. As the quality of caregiving and parental well-being are known to associate with children development (Roggman et al., 2013; Totsika et al., 2020), providing tailored parental support during tele-medicine interventions should be considered as a key element of the intervention itself. Notably, tele-medicine interventions can also facilitate the direct provision of rehabilitation programs for older children that can benefit from the on-line availability of therapeutic games and apps which can also provide the therapists with an ongoing monitoring of the rehabilitation journey (Corti et al., 2018; Oldrati et al., 2020).

**Figure 2:**
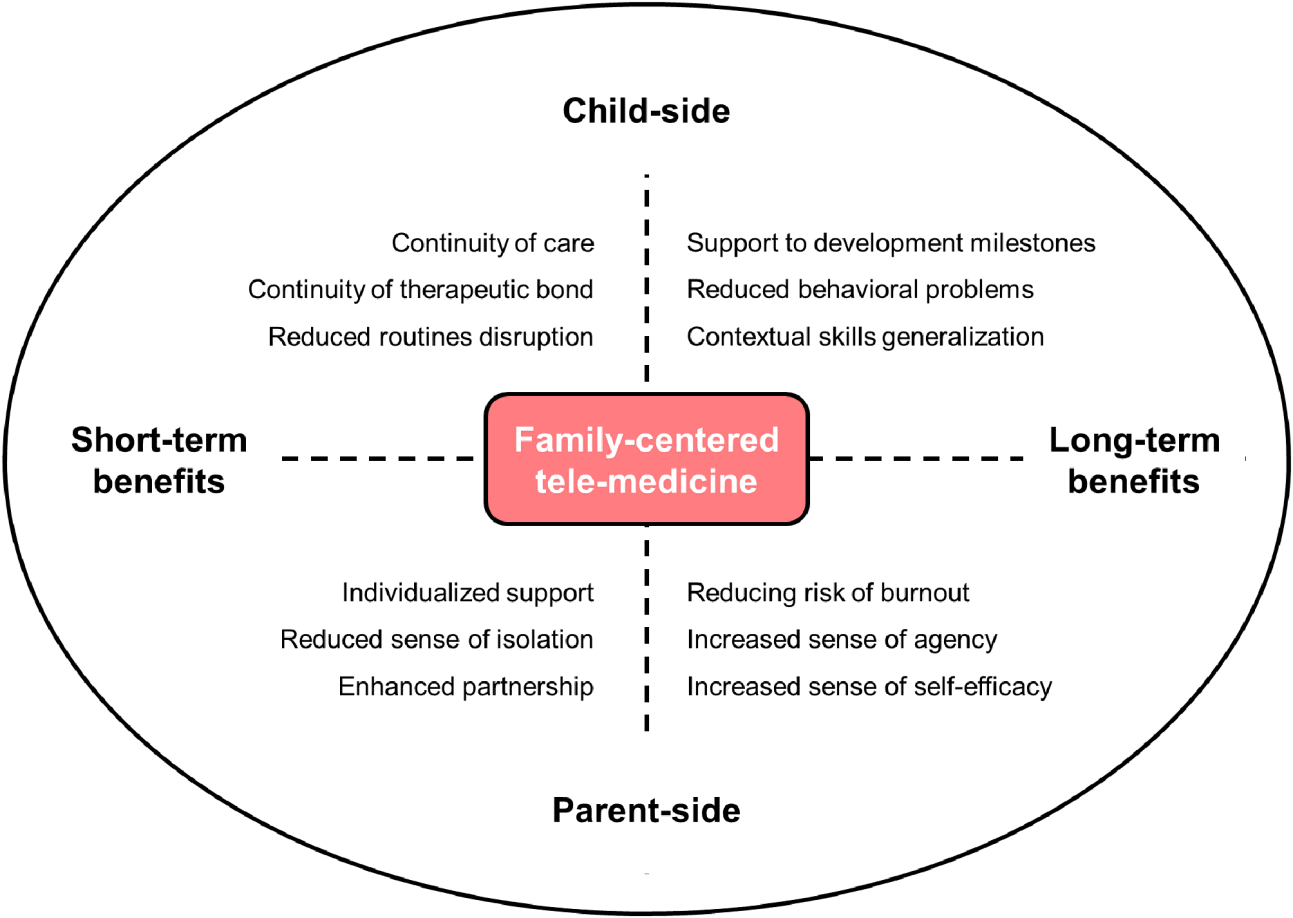
The potential benefits of family-centered tele-medicine for parents of children with neurodevelopmental disabilities. The horizontal axis represents the time frame of benefits, whereas the vertical axis represents the different positive effects for the parents and the child.

Such a family-centered approach to tele-medicine in child neuropsychiatry is warranted to provide positive effects for both children and families (Figure 2). On the child-side, by promoting continuity of care the risk of disrupting daily and weekly routines is reduced and children can maintain social and affective contacts with their therapists and specialists. Moreover, on-line rehabilitation programs can support the achievement of developmental milestones in behavioral, cognitive and social abilities (Langkamp et al., 2015; McConnochie et al., 2015; Knutsen et al., 2016). As parents' role in facilitating the rehabilitation interventions is much more prominent in online interventions, they can be more actively engaged by therapists (Myers et al., 2017; Ray et al., 2017). Additionally, parents can receive psychological and educational support to meet their own needs and those of their children (Harper, 2006; Hinton et al., 2017). Finally, by actively engaging in remote therapies and rehabilitation sessions they can increase their sense of agency and self-efficacy in parenting.

## Insights From an Ongoing Italian Family-Centered Tele-Medicine Program During the Covid-19 Lockdown

Soon after the start of the lockdown phase in Italy, a family-centered tele-medicine program—namely, the Engaging with Families in On-line Rehabilitation of Children during the Epidemic (EnFORCE) program—was launched at the Child Neurology and Psychiatry Unit of the IRCCS Mondino Foundation, in Pavia. This hospital is located in the primary hotspot of Covid-19 spread in Italy and receives patients from the surrounding Lombardia region as well as from other regions. The EnFORCE program was aimed at providing families of children with neurodevelopmental disabilities that were already enrolled in outpatient activities with an immediate tele-medicine support, reducing the risk of emotional distress and psychological burnout during the lockdown phase. The majority of rehabilitation interventions that were planned or ongoing for the physical setting were modified in order to be at least partially delivered on-line, engaging parents and promoting a positive rehabilitation partnership with therapists and healthcare professionals. The primary aim was promoting continuity of care for children; nonetheless, a secondary goal was providing emotional and psychological support to the parents during an unprecedented challenging time. More than 80 families were enrolled in the study. The preliminary findings suggest that although none of the included parents had been positive to Covid-19, half of them were living in a high-contagion rate geographical area and up to 20% had experienced the loss of a loved one. Concerns for the child health and the continuity of rehabilitation programs were among the greatest sources of emotional distress for these parents, confirming the psychological burden of lockdown in families of children with disability. At the present moment, the intervention is ongoing and data on the reduction of parents' psychological burden are not available. Nonetheless, all the eligible families accepted to be enrolled in the intervention with almost 100% of participation to the on-line sessions, which suggests that the EnFORCE program was well-received by parents.

## Challenges for Family-Centered Tele-Medicine

Tele-medicine implies also some specific critical aspects to manage. First, relevant challenges regard the security and safety of data management and families' privacy. Tele-medicine intervention require the shift of data storage and access on cloud services. While this has the advantage of being convenient (e.g., complete patient history can be available in real-time) and cost-effective (Esposito et al., 2018), cloud deployments in healthcare industry are vulnerable to threats posed by both external attacks and service providers. Cryptographic systems, such as block-chain technology, offer flexible and efficient solutions (Guo et al., 2019). Moreover, policies for data management security and protection differ between countries. The regulation concerning data ownership and datacentre locations can be conflicting in different states and even within the European Union there may be different degrees of control and limitations to healthcare data storage, sharing and management (Currie and Seddon, 2014; Esposito et al., 2018). Second, although smartphones, computers and tablets are thought to be generally available to most of the families, demographic data only partially support this common sense view. A recent survey conducted in the biennium 2018–2019 by the Italian National Institute of Statistics (ISTAT)^1^ revealed that approximately the 33% of families had no computer or tablet at home; this estimates was lower (14%) for families with at least one child. Only 22% of families had a one-to-one member-device ratio and families with low socio-economic status were especially lacking the availability of computers and tablets. With the growing digitalization of healthcare (Mishon et al., 2020; Moro Visconti and Morea, 2020), the availability of technological devices in home environments becomes a key requirement for accessing healthcare services and governments need to provide adequate economic support to promote equality and reduce socio-economic disparities. Finally, the rehabilitation materials and methods need to be at least partially adapted to the tele-medicine settings. This adaptation also regards the specialist-parent relationship. Indeed, clinicians usually have a primary role in the direct management of the rehabilitation activities, while parents are often left on the bench. In tele-medicine settings, the therapists are outside of the family physical space and the development of an optimal partnership with the parents becomes both a key goal and a crucial proxy for the success of the rehabilitation program itself.

## Conclusions

The COVID-19 pandemic is asking specialists in the field of child neuropsychiatry and rehabilitation to at least partially shift to tele-medicine programs. Nonetheless, this unprecedented period of healthcare and socio-economic crisis can also become an opportunity (Provenzi and Barello, 2020). Indeed, by improving our ability to use innovative technologies to respond to the special healthcare needs of children with disability and their families, we may proceed forward to build more inclusive societies and smarter healthcare systems. In other words, tele-medicine strategies in developmental neuropsychiatry should not be considered as an emergency response only. Rather, as tele-medicine makes healthcare services accessible by underserved and resource-constrained communities (Andreassen and Dyb, 2010; Khilnani et al., 2020), this is an unmissable occasion to create new bridges to reduce inequalities in healthcare for children and families. Even when we will be able to consider this emergency passed, tele-medicine solutions are warranted to be a positive heritage of our virtuous response to the current pandemic.

## Author Contributions

LP conceived the initial draft of this work. SG drafted the final version of the work. RB provided scientific supervision. All authors contributed to this work and agreed on the submission of the final manuscript version.

## Mondino EnFORCE Group Members

Valentina Aramini, Ilaria Baschenis, Angela Berardinelli, Laura Bernasconi, Luca Capone, Camilla Caporali, Adriana Carpani, Stefano Cassola, Matteo Chiappedi, Raissa Francesca Costantino, Erika Dargenio, Valentina De Giorgis, Federica Ferro, Alice Gardani, Antonella Luparia, Chiara Magni, Martina Mensi, Cecilia Naboni, Simona Orcesi, Elena Saligari, Sabrina Signorini, Martina Tosi, Valeria Vacchini, Costanza Varesio, Elena Vlacos, Martina Zanaboni.

## Conflict of Interest

The authors declare that the research was conducted in the absence of any commercial or financial relationships that could be construed as a potential conflict of interest.

## Footnotes

^1^Data retrieved here: https://www.istat.it/it/archivio/240949.
